# Supplementary material for: Comparative multiomics analysis of cell physiological state after culture in a basket bioreactor
Source: Sci Rep. 2022 Nov 23;12:20161. doi: 10.1038/s41598-022-24687-4 (PMC9686226; doi:10.1038/s41598-022-24687-4)

1:437942:438074:-@NW_023666047.1:436365:436668:-@NW_023666047.1:436016:436062:-@NW_023666047.1:4

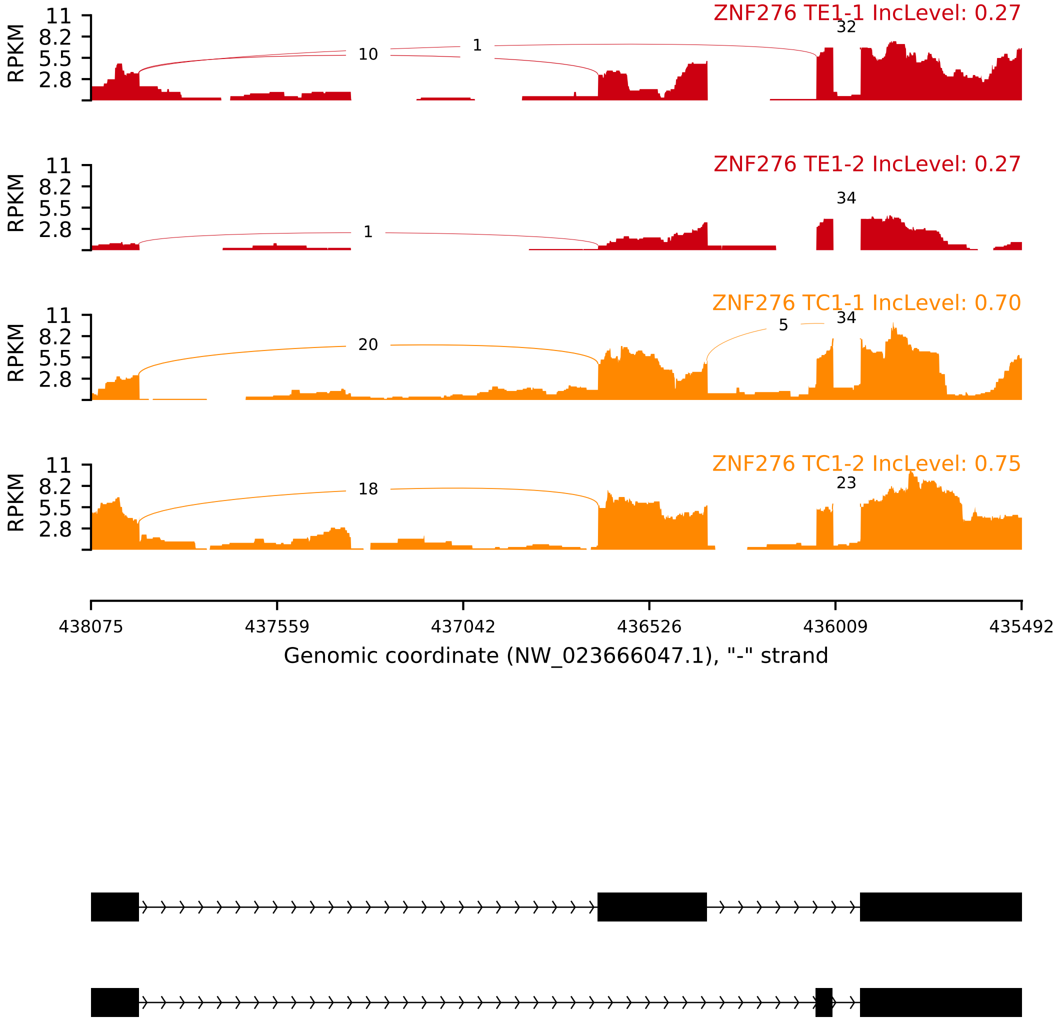

Supplement: Supplementary file 1 — Supplementary Information 1. [file 41598_2022_24687_MOESM1_ESM.zip › raw data/Transcriptomic raw data/7.AS/2.dasplot/TE1vsTC1/MXE/9_ZNF276_NW_023666047.1_437942_438074_-@NW_023666047.1_436365_436668_-@NW_023666047.1_436016_436062_-@NW_023666047.1_435490_435939_-.pdf]
